# Supplementary material for: Prediction of Lupus Classification Criteria via Generative AI Medical Record Profiling
Source: BioTech (Basel). 2025 Mar 6;14(1):15. doi: 10.3390/biotech14010015 (PMC11940096; doi:10.3390/biotech14010015)
Supplement: Supplementary file 1 [file biotech-14-00015-s001.zip › biotech-3455351-supplementary.pdf]

BioTech

MDPI

Supplementary Materials

Supplementary Information Tables S1, S2.

Prediction of Lupus Classification Criteria Via Generative AI Medical Record Profiling.

Manuscript ID: biotech-3455351. (Nair et al.)

Table S1. Statistics for genAI prediction of ACR criteria, compared to clinical assessments by practicing rheumatologists. Comparisons are over 27 patients with full clinical criterial reports.

Criteria	Accuracy	TP	FP	FN	TN	PPV	NPV	Sens	Spec
**1. Malar Rash**	0.78	0	1	5	21	0.00	0.81	0.00	0.95
**2. Discoid Rash**	1	0	0	0	27	n/a	1.00	n/a	1.00
**3. Photosensitivity**	0.7	3	1	7	16	0.75	0.70	0.30	0.94
**4. Oral Ulcers**	0.63	1	0	10	16	1.00	0.62	0.09	1.00
**5. Nonerosive Arthritis**	0.59	8	10	1	8	0.44	0.89	0.89	0.44
**6. Pleuritis or Pericarditis**	1	0	0	0	27	n/a	1.00	n/a	1.00
**7. Renal Disorder**	0.78	0	6	0	21	0.00	1.00	n/a	0.78
**8. Neurologic Disorder**	0.93	0	2	0	25	0.00	1.00	n/a	0.93
**9. Hematologic Disorder**	0.78	2	3	3	19	0.40	0.86	0.40	0.86
**10. Immunologic Disorder**	0.56	4	12	0	11	0.25	1.00	1.00	0.48
**11. Antinuclear Antibody**	0.81	16	2	3	6	0.89	0.67	0.84	0.75
**Total Criteria**	0.78	34	37	29	197	0.48	0.87	0.54	0.84
**Neyman 95% C.I.**	0.081	9.9	8.4	6.8	14.6	0.116	0.044	0.123	0.047

Table S2. Representative examples of genAI-selected evidence for determination of different SLE criteria. Examples of false positive determinations are also appended (reverse colors). Protected identifiers (names, locations and dates) have been replaced with "**".**

Criterion type	genAI verdict	genAI evidence (verbatim from medical records)
1. Malar Rash	Completely Met	"Patient continues to have malar rash" (****)<br>"Skin malar erythema - butterfly rash" (****)'
1. Malar Rash	Completely Met	Patient complains of rash on her face, she has photosensitivity and cannot expose to sun for longer than 5 minutes.'
2. Discoid Rash	Completely Met	"She says she had a rash on her face by her right eyebrow. Says eyebrow is now non-existent. She went to a dermatologist and was told she trichotillomania. She says it was very hard to talk about b/c of this. She felt **** the skin tissue in her forehead was thickened. She

		also notes recurrences of ingrown hairs in a cluster. The area will itch SO she had rubbed and pulled at hair there but she says that was not from anxiety or intenitial." (****)'
3. Photosensitivity	Completely Met	"She gets sunburn very easily, denies any skin rashes." (****)'
3. Photosensitivity	Completely Met	"Integumentary: Positive, Photosensitivity." (****)'
4. Oral Ulcers	Completely Met	"She was complaining of fatigue, joint been an oral ulcers." (****)'
4. Oral Ulcers	Completely Met	"She complains of some more frequent mouth sores, dry eyes, and dry mouth." (****)<br>"She has had mouth sore, has been unable to wear her dentures." (****)'
5. Nonerosive Arthritis	Completely Met	"Right shoulder is tender, not swollen. Left wrist is tender, not swollen. Right 3-5 MCPs are tender, with swollen 2nd and 3rd. Right 2-5 PIPs are tender, not swollen. Left 1-5 MCPs are tender, with swollen 2nd and 3rd. Left ankle is tender and swollen." (****)<br>"She has pain in the right shoulder, bilateral hands, and the left ankle. There is swelling of the left ankle." (****)'
5. Nonerosive Arthritis	Completely Met	"Patient said her symptoms started after she had COVID vaccine. At that time she was having chest pain, fatigue, migraines and migratory joint pain." (****)<br>"Patient complains of bilateral hip pain. On exam she was found to have palpable tenderness over bilateral trochanteric bursa." (****)'
5. Nonerosive Arthritis	Completely Met	"Arthralgias, Joint pain, Joint stiffness" (****)<br>"Cervical spine - mild pain w/ motion. Thoracic spine - mild pain w/ motion. Lumbar spine - Range of motion: mild pain w/motion." (****)<br>"Shoulder - Left: Range of motion: mild pain w/ motion, Right: Range of motion: mild pain w/ motion. Hands - Left: Range of motion: mild pain w/ motion, Right: Range of motion: mild pain w/ motion. Knee - Left: Range of motion: mild pain w/ motion, Right: Range of motion: mild pain w/ motion. Foot/ankle - Left: Range of motion: mild pain w/ motion, Right: Range of motion: mild pain w/ motion." (****)'
6. Pleuritis or Pericarditis	Completely Met	"She relates that she began to experience chest discomfort as well as intermittent dyspnea **** and was evaluated by Cardiology at that time without any significant

		findings on stress test, echocardiogram, or heart catheterization per patient." (****)'
6. Pleuritis or Pericarditis	Completely Met	"He suffered an episode of pericarditis in **** with normal cardiac catheterization at that time." (****)'
6. Pleuritis or Pericarditis	Completely Met	"Chest pain consistent with pleurisy. Now resolved following prednisone taper." (****)<br>"Chest pain, especially when taking a deep breath or when lying down." (****)'
7. Renal Disorder	Completely Met	"Recent labs completed on **** included an essentially unremarkable CBC, CMP, normal CRP, C3, C4, negative dsDNA, and abnormal UA." (****)<br>"Recent labs completed on **** included an essentially unremarkable CMP, CBC showed depressed WBC at 2.9, C3, C4, CRP were normal, dsDNA was negative, and UA showed 1 + protein." (****)'
7. Renal Disorder	Completely Met	Increase in proteinuria on lab evaluation, he was scheduled to see Nephrology, but not for several months. Repeated urinalysis to make sure that protein-to-creatine ratio was not increasing.'
7. Renal Disorder	Completely Met	"UA revealed proteinuria with protein:creatinine was 0.630." (****)<br>"Recent labs completed on **** included a CBC with mild leukopenia, CMP was unremarkable, normal ESR, CRP, C3, C4, hyaline casts of 0-5, and 1 + proteinuria." (****)'
8. Neurologic Disorder	Completely Met	"She had a seizure, had lab work done which reportedly showed SLE." (****)<br>"She had seizures in ****, none ****." (****)'
8. Neurologic Disorder	Completely Met	"She had experienced a severe HA **** and underwent a brain MRA as well as a head CT without any significant findings, results again requested." (****)<br>"She relates that she developed a severe HA ~2 weeks ago and recently underwent a brain MRA as well as a head CT, results currently pending." (****)'
8. Neurologic Disorder	Completely Met	"Patient has a history of grand mal seizure disorder currently treating with gabapentin." - ****'
9. Hematologic Disorder	Completely Met	"Recent labs completed on **** included an essentially unremarkable CMP, CBC showed depressed WBC at 2.9, C3, C4, CRP were normal, dsDNA was negative, and UA showed 1 + protein." (****)<br>"Recent labs completed on **** included an essentially

		unremarkable CMP, CBC showed mild normocytic anemia, and CRP was normal." (****)'
9. Hematologic Disorder	Completely Met	Recent labs completed on **** included a CBC with worsening normocytic anemia
10. Immunologic Disorder	Completely Met	"AVISE panel was positive with an ANA at 1:320 and anti-glycoprotein1 Ab." (****)'
10. Immunologic Disorder	Completely Met	"Double-stranded DNA is elevated 1: 640" (****) "Complement C3 is low at ****, down from 45 (****), Complement C4 is low at 6, stable from prior study" (****)'
10. Immunologic Disorder	Completely Met	"ANA SCREEN, IFA POSITIVE" on ****. "ANA TITER 1:80 H" on ****. "DNA AB (DS) CRITHIDIA, IFA NEGATIVE" on ****. "CARDIOLIPIN AB (IGA) <2.0", "CARDIOLIPIN AB (IGG) <2.0", "CARDIOLIPIN AB (IGM) <2.0" on ****.'
11. Antinuclear Antibody	Completely Met	"ANA SCREEN, IFA ,,POSITIVE ,NEGATIVE" - Lab Result dated **** "ANA TITER ,,1:640 H ,titer" - Lab Result dated **** "ANA PATTERN ,,Nuclear, Dense Fine Speckled" - Lab Result dated ****
11. Antinuclear Antibody	Completely Met	"Her AVISE panel was positive for lupus." (****) "Labs done previously showed elevated titers to ANA, SSA, and rheumatoid factor." (****)
12. Previous Diagnosis	Completely Met	"*** is a pleasant ****. female with history of SLE, possible RA overlap, ****, and OA." (****) "*** is a pleasant ** female with history of SLE/possible RA overlap as well as APLA and OA." (****, ****, ****, ****, ****, ****, ****, ****)
10. Immunologic Disorder	Completely Met	Last labs completed on 9/20/21 included a normal C3, C4, and negative dsDNA Ab, Recent labs completed on 5/31/23 included an essentially unremarkable CBC, CMP, and a normal C3, C4, CK, negative dsDNA, and UA showed no blood or protein.
3. Photosensitivity	Completely Met	denies any rashes related to photosensitivity as he does not go out in the sun.'
4. Oral Ulcers	Completely Met	"Patient denies any oral/pharyngeal ulcers" (****)'
